# Nurses’ knowledge and practice regarding mixing medications with food: a multicenter cross-sectional study from a developing country

**DOI:** 10.1186/s41043-023-00396-0

**Published:** 2023-06-05

**Authors:** Marah A. Daibes, Rawan I. Qedan, Samah W. Al-Jabi, Amer A. Koni, Sa’ed H. Zyoud

**Affiliations:** 1 Department of Clinical and Community Pharmacy, College of Medicine and Health Sciences, An-Najah National University, Nablus, 44839 Palestine; 2 Division of Clinical Pharmacy, Department of Hematology and Oncology, An-Najah National University Hospital, Nablus, 44839 Palestine; 3 Poison Control and Drug Information Center (PCDIC), Faculty of Medicine and Health Sciences, An-Najah National University, Nablus, 44839 Palestine; 4 Clinical Research Center, An-Najah National University Hospital, Nablus, 44839 Palestine

**Keywords:** Knowledge, Practices, Food, Medication, Modification, Mixing, Crushing, Nurses

## Abstract

**Background:**

Different pharmaceutical characteristics of the dosage form (DF) have a direct effect on how easily oral solid medicine is swallowed. The practice of crushing tablets or opening the capsule occurs daily in the hospital, and most nurses are unknowledgeable regarding these issues. Coadministration of medications with food can cause changes in drug absorption and lead to an alteration in gastrointestinal motility, which can cause an unexpected effect on the dissolution and absorption of the drug. Therefore, this study aimed to investigate nurses' knowledge and practices regarding the mixing of medications with food or drink in Palestine.

**Methods:**

From June 2019 to April 2020, a cross-sectional study was conducted, encompassing nurses working in government hospitals across various districts of Palestine. The data were collected through face-to-face interviews, using questionnaires that assessed nurses' understanding and implementation of mixing medications with food. The sampling method employed was convenience sampling. To analyze the gathered information, the Statistical Package for the Social Sciences version 21 (IBM-SPSS) was utilized.

**Results:**

A total of 200 nurses participated in the study. The data show a significant difference between the median knowledge scores according to the department of work (*p* < 0.001). The highest median [interquartile] knowledge score of 15 [12–15] was found for nurses working in the neonatal intensive care unit. In addition, nurses in the pediatric ward and the men’s medical ward had high scores of 13 [11.5–15] and 13 [11–14], respectively. In general, the results show that 88% of nurses modified oral DF prior to administration to patients. Regarding the type of food used, mixing medicine into juice was the most common procedure performed by nurses (approximately 84%); 35% of nurses used orange juice to mix with medicine. The most common reason for crushing was to administer medications to patients with a nasogastric tube (41.5%). In regard to medications, aspirin was the most frequently used drug that was crushed by the nurses (44%); however, 35.5% of nurses did not feel sufficiently trained to carry out this practice. Concerning the sources of information, 58% of nurses usually asked pharmacists for information about medications.

**Conclusions:**

The results of this study show that crushing and mixing medications with food is common among nurses, and most nurses are unaware of the dangerous effect of this practice on patient health. Pharmacists, as medication experts, should participate in sharing knowledge about unnecessary crushing situations or when crushing should be avoided and try to find an alternative, when available, to aid administration.

## Background

Patient acceptability of medication products is a cornerstone of the development of dosage forms (DFs) and the prescription of medicines. However, older adults and children differ from other age groups and require careful consideration, particularly regarding medication acceptability [1]. Different pharmaceutical characteristics of DFs, such as the shape, size, and surface texture of the tablet, have important effects on how easily an oral solid DF of medicine is swallowed and passed throughout the pharynx and oesophagus [2].

Oral medication administration seems to be the safest and simplest way of treating patients. Unfortunately, patients who have problems with swallowing oral medication (dysphagia) or in the use of oral medications in general may have problems in finding suitable pharmaceutical DF. Therefore, they usually need to crush tablets or open capsules (CT/OC), which is, for many medications, an unlicensed procedure [3, 4]. Modifying solid oral medication has an impact on the safety, quality, and efficacy of the medication and may cause adverse effects [5]. Unfortunately, modification of DF can affect the chemical or physical stability of the drug or drug bioavailability, leading to increased toxicity or reduced efficacy and interfering with patient outcomes [6, 7]. Moreover, the manufacturer will not bear any responsibility for any harm to the patient after changing a pharmaceutical DF [8].

Not all oral medications can be split. Moreover, splitting (cutting in half) or crushing pharmaceutical DF-like extended-release (ER) tablets may be harmful or dangerous in some situations. In addition to older adults with swallowing difficulty crushing their medications for easier administration, nurses also crush medications for patients with a feeding tube. If these medications are not intended for crushing, this procedure can be problematic and may be harmful. Patients should be advised not to crush or split the medication without checking if it is suitable by discussing it with a pharmacist or other healthcare providers [3, 9]. Administration of crushed medication may lead to therapeutic failure, patient injury, or drug toxicity. Drugs labelled controlled-release (CR), sustained-release (SR), modified-release (MR), or extended-release (ER) should be swallowed whole and should not be crushed or split. Crushing this DF will mean the patient receives the entire dose of medication at once, not over a prolonged period. This leads to medication toxicity, with life-threatening outcomes. Other medications of concern have narrow therapeutic windows, such as phenytoin, digoxin, and sodium valproate [10, 11].

In fact, the practice of crushing tablets or opening the capsule occurs daily in the hospital, and the percentage of nurses who adopted this practice is approximately 28%, with a high percentage of 67% in the geriatric wards [1]. Furthermore, this practice is prevalent in pediatric units, where the majority of nurses are unknowledgeable regarding important issues of mixing drugs with food, such as dissolution and stability, and the clinical consequences of this action [12]. Similarly, another study reported a high percentage (87%) of nurses who modified drugs before administration and found that yoghurts and fruit juice were frequently used [13]. Furthermore, a study showed that nurses were unaware of drug administration through enteral feeding tubes, but their knowledge improved significantly after training by clinical pharmacists; that is, the percent of knowledge of the mixed medications substantially increased from 23.3% to 55.3% [14]. Another investigation assessed the knowledge and practices of nurses and concluded that basic knowledge of medication administration through enteral feeding tubes was insufficient and nurses had poor practice in this regard [15]. In a previous cross-sectional study, crushing tables by nurses was among the most common drug modifications, and swallowing difficulty was the most common reason [16]. The high percentage of this common practice among nurses and the detrimental effects of unawareness of drug modification encouraged us to conduct this study.

This topic seems neglected in the literature; to the best of our knowledge, few articles highlight the knowledge and practices among nurses regarding the mixing of drugs with food; however, most of these publications assess the aspect of drug-food interactions and were carried out in pediatric nurses [1, 6, 12–14, 17–19]. In fact, little information is published regarding this topic in nurses from geriatric wards and intensive care units, where the nasogastric tube is used, and there are no available data in other hospital wards. Furthermore, evidence that confirms this practice is widely available [1, 12, 13, 16], but published data on this common practice in Palestine are lacking. The current study tries to fill this gap, and therefore, the researchers want to evaluate the knowledge and practices regarding the mixing of oral medications with food or drink and examine the impact of nurse characteristics on the level of knowledge.

## Methods

### Study design and setting

This was a cross-sectional study designed to measure nurses’ knowledge and practices about mixing medications with food. The data were collected between February and April 2019.

Palestine consists of two geographical areas: The West Bank and the Gaza Strip. According to the Palestinian health annual report 2021, there are approximately 5.29 million inhabitants in Palestine, 59.6% in the West Bank and 40.4% in the Gaza Strip [20]. There are three regions in the West Bank:

- The North Region is divided into six governorates: Jenin, Tulkarm, Nablus, Qalqilya, and Tubas.
- The Middle Region is divided into three governorates: Jerusalem, Ramallah, Salfit, and Jericho.
- The South Region is divided into two governorates: Bethlehem and Hebron.

The current study was carried out in the West Bank of Palestine. We chose the West Bank because it is difficult to reach the Gaza Strip due to its boundaries. We collected information from all government secondary and tertiary hospitals in the northern West Bank. A list of government hospitals' names and addresses was obtained from the Ministry of Health (MOH). Based on these lists, we visited the following West Bank governorates: Nablus, Jenin, Qalqilya, Tulkarm, Salfit, and Tubas [21]. These hospitals provide several services and have different disciplines, including pediatric and geriatric units.

### Sample size calculation

In 2019, the Palestinian Health Information Centre gathered data indicating that there were a total of 1822 nurses employed in government hospitals located in the West Bank [22]. For the purpose of this study, a specific group of hospitals was selected, and within those hospitals, there were 769 nurses included in our analysis [22]. To ensure the reliability of our findings, we calculated the required sample size using the Raosoft sample size calculator [23], aiming for a 95% confidence level and a 5% error margin. The calculator indicated that a minimum sample size of 257 nurses was necessary.

### Sampling procedure

In this study, the population was chosen from nurses in government hospitals in the northern region of the West Bank of Palestine, according to data taken from the Palestinian Health Information Center. Nurses from different hospitals were invited to participate in the study using a convenience sampling technique. We reached the nurses in each department during their morning shift, and they were interviewed face-to-face.

### Inclusion and exclusion criteria

In 2019, the total number of registered nurses employed in government hospitals and health care units in the West Bank was 2613 [22]. The population was selected from nurses employed in northern government hospitals. We included all six government hospitals in northern Palestine: Al-Watani Hospital, Martyr Dr. Thabet Governmental Hospital, Rafidia Surgical Hospital, Tubas Turkish Hospital, Darwish Nazzal Hospital, and Jenin Government Hospital.

- The inclusion criteria were as follows: a registered nurse in the Palestinian Ministry of Health (MOH), with at least a certificate or higher degree of qualification (master’s degree or PhD) and employed in a government hospital. All working nurses, regardless of their wards, were able to participate. We included nurses with a diploma because there are no restrictions in Palestine that prevent them from preparing medications without assistance.
- The exclusion criteria were nurses who refused to participate in our study.

### Data collection instrument form

The semistructured questionnaire on the knowledge and practices of healthcare professionals regarding the mixing of medications with food, especially nurses, has been developed in previous studies [1, 7, 12–14, 24–27], which were carried out in different places, including France, the Netherlands, the UK, Iran and Palestine.

### Demographics

The questionnaire contains five sections. The first was the demographic section, which included issues relating to age, gender, work years, marital status, region of residence, level of education, place of graduation, year of graduation, experience, and training background or specialty.

### Nurses’ practices of mixing medications with foods

The second section is divided into three parts. The first evaluated how frequently the nurses modified the dosage form and how they dealt with this modification by adding crushed tablets or capsules to food or drinks or inversely. This was based on a review of the literature, the experience of researchers and the consultation of clinical experts. This part consisted of seven questions, each answered yes or no. The second part determined how often each kind of food (banana, milk, juice, etc.) was used to mix with crushed medicine. The list contained seven types of foods or drinks, and the nurses could add other kinds. They were asked how frequently food was mixed with medicine (never, rarely, sometimes, often, or very often). The third part was a list of drugs (analgesics, antihypertensives, antiplatelets, antibiotics, corticosteroids, diuretics, antilipidemic, and gastric acid-reducing medications), and the nurses chose from it what medication they commonly crushed and mixed with food before administering to the patients to determine the most common drugs crushed and mixed with food by nurses. The answers to these questions are coded as yes or no.

### Reasons for dosage modifications

The third section determined the reasons for the modification of the dosage form of the drug in hospitals and consisted of five potential reasons based on the literature review and clinical expert consultation (never, rarely, sometimes, often, or very often) [1, 7, 12–14, 24–27].

### Nurses’ knowledge regarding mixing medications with foods and sources of information.

The fourth section was divided into two parts. The first evaluated nurses’ knowledge about mixing drugs with food, food-drug interactions, and chemical/physical stability of drugs after modification and whether they were sufficiently prepared to carry out this procedure or reported every time they modified the dosage form. It consisted of 18 questions, answer yes, no, or I don’t know. No cut-off point was used to determine knowledge. Instead, the total median score was calculated, with a higher score indicating greater knowledge, and then compared between all variables.

The second part was a multiple-choice question about the nurses’ sources of information about splitting or crushing tablets or capsules; they could choose more than one.

### Transparency of the dosage modification process

The fifth section was about the transparency of the modification procedure, asking about receiving permission from patients to mix the medicine with food or if they asked them about their favorite food to use for mixing with medication. It was also asked if the mixing procedure was written on nurses’ notes or mentioned by the prescriber. It consisted of seven questions answered never, rarely, sometimes, often, or always.

At the end of the interview, we provided nurses with an educational orientation on incorrect answers that may harm the patients.

### Ethical approval

Before the start of this study, authorisation in all aspects, including access to and use of the information in our study procedure, was obtained from the *Institutional Review Board (IRB) of An-Najah National University* [approval number: (1) June 2018], MOH, and local health authorities. Before filling out the questionnaire, all parts of the study, including study instruments, objectives, privacy, and confidentiality (all information will be used only for research purposes), were completely explained to the participants, and verbal consent was received from them.

### Study rigor

The questionnaire was reviewed by consensus by a panel of three experts in the field drawn from academia for its content validity (one expert in clinical pharmacology and two experts in clinical pharmacy). The questionnaire was originally created in English and subsequently translated into Arabic by two highly skilled healthcare professionals (SHZ and SWA), who possess vast expertise in health survey design and are fluent in both languages. All experts stated that the questions adhered exclusively to the study’s objectives. A pilot study with 25 participants from all included hospitals was conducted to test the instrument of our study to ensure readability, estimate the time and then adjust the data collection form (questionnaire) as needed.

### Statistical analysis

Data entry and analysis were performed using the IBM Statistical Kit for Social Sciences version 21 program (IBM-SPSS). Medians (interquartile ranges [IQRs]) were used to present continuous variables, while frequencies (percentages) were used for categorical variables. The normality of each variable was assessed using the Kolmogorov‒Smirnov test. To evaluate median differences between groups, either the Kruskal‒Wallis or Mann‒Whitney tests were employed. Statistical significance was denoted by a *p* value of less than 0.05.

## Results

### Sociodemographic data

This study was a cross-sectional study conducted among 260 nurses working in six government hospitals in the West Bank region of Palestine. However, approximately 60 nurses were excluded according to the exclusion criteria. Therefore, the final analysis was performed on 200 samples. As Table 1 indicates, most nurses (approximately 75%) were married, and females (approximately 57%) divided equivalently between hospitals. A total of 66% of the sample had a bachelor’s degree in nursing, and the vast majority of them (approximately 87%) were younger than 40 years. Furthermore, 45.5% of the participating nurses had 5–10 years of work experience. Nurses were distributed among several departments, most of them in the internal medicine department (approximately 29%), pediatrics (16.5%) and ICU (14.5%).

**Table 1:** Demographics of the respondents (*n *= 200)

Variable	Frequency (%)
*Gender*	
Male	86 (43)
Female	114 (57)
*Marital status*	
Single	50 (25)
Married	150 (75)
*Hospital*	
Rafidia Surgical Hospital	33 (16.5)
Al-Watani Hospital	33 (16.5)
Jenin Government Hospital	33 (16.5)
Tubas Turkish Hospital	33 (16.5)
Martyr Dr. Thabet Thabet Governmental Hospital	33 (16.5)
Darwish Nazzal Hospital	35 (17.5)
*Work department*	
Emergency room	1 (0.5)
ICU	29 (14.5)
NICU	15 (7.5)
Pediatric	33 (16.5)
MMW	27 (13.5)
WMW	31 (15.5)
Bone	13 (6.5)
Nephrology	21 (10.5)
Surgery	21 (10.5)
Wound	4 (2)
Delivery	4 (2)
General	1 (0.5)
*Years of work*	
Less than 3 years	41 (20.5)
5–10 years	91 (45.5)
More than 10 years	68 (34)
*Age (year)*	
20–29	80 (40)
30–39	95 (47)
40–49	20 (10)
50–59	5 (2.5)
*Education level*	
Diploma	57 (28.5)
Bachelor’s degree	132 (66)
Master’s degree	11 (5.5)

*ICU* Intensive care unit, *NICU* Neonatal intensive care unit, *MMW* Men’s medical unit, *WMW* Women’s medical ward

### Self-knowledge score and sociodemographic variables

Table 2 shows a significant difference between participants according to work department (*p* value < 0.001). The highest median [interquartile] score of 15 [12–15] was found for nurses working in the neonatal intensive care unit. Additionally, nurses in the pediatric ward and the men’s medical ward had high scores of 13 [11.5–15] and 13 [11–14], respectively. The wound and delivery departments had a high score, but there were only four participants in these departments. No significant differences were observed in marital status, hospital, years of work, age, or educational level. For example, although the 40–49 age group has the highest median compared to other groups, the difference was not significant.

**Table 2:** Distribution of the knowledge score by sociodemographic variables (*n *= 200)

Variable	Percentage *n* (%)	Median [*Q*1–*Q*3]	Mean rank	*P* value
*Gender*				
Male	86 (43)	12 [9.7–4]	90.6	**0.035**^**a**^
Female	114 (57)	13 [11–15]	107.97	
*Marital status*				
Single	50 (25)	12 [10–15]	102.16	0.814^a^
Married	150 (75)	12 [11–14]	99.95	
*Hospital*				
Rafidia Surgical Hospital	33 (16.5)	13 [12–15]	124.15	0.171^b^
Al-Watani Hospital	33 (16.5)	12 [10–14]	97.44	
Jenin Government Hospital	33 (16.5)	12 [10–13.5]	87.89	
Tubas Turkish Hospital	33 (16.5)	12 [9.5–14]	97.5	
Martyr Dr. Thabet Thabet Governmental Hospital	33 (16.5)	12 [11–14.5]	101.83	
Darwish Nazzal Hospital	35 (17.5)	12 [11–14]	94.54	
*Work Department*				
Emergency room	1 (0.5)	–	195.5	** < 0.001**^**b**^
ICU	29 (14.5)	11 [8–12]	64.83	
NICU	15 (7.5)	15 [12–15]	138.4	
Pediatric	33 (16.5)	13 [11.5–15]	119.62	
MMW	27 (13.5)	13 [11–14]	101.7	
WMW	31 (15.5)	11 [7–14]	78.94	
Bone	13 (6.5)	12 [10–14.5]	100.54	
Nephrology	21 (10.5)	12 [11–14]	94.64	
Surgery	21 (10.5)	12 [11–14]	107.26	
Wound	4 (2)	13.5 [13–14.75]	134.63	
Delivery	4 (2)	14.5 [11.75–15.75]	138.88	
General	1 (0.5)	–	167.00	
*Years of work*				
Less than 3 years	41 (20.5)	13 [11–15]	111.39	0.310^b^
5 years—10 years	91 (45.5)	12 [10–14]	94.91	
More than 10 years	68 (34)	12 [11–14]	101.41	
*Age (year)*				
20–29	80 (40)	12 [10–14.75]	101.61	0.674^b^
30–39	95 (47)	12 [10–14]	96.6	
40–49	20 (10)	13 [11–14.75]	113.68	
50–59	5 (2.5)	12 [11–14.5]	104.10	
*Education level*				
Diploma	57 (28.5)	12 [10–14.5]	99.29	0.966^b^
Bachelor’s degree	132 (66)	12 [11–14]	100.73	
Master’s degree	11 (5.5)	12 [11–14]	104.05	

^a^Statistical significance of the differences was calculated using the Mann‒Whitney U test

^b^Statistical significance of the differences calculated using the Kruskal‒Wallis test

*ICU* Intensive care unit, *NICU* Neonatal intensive care unit, *MMW* Men’s medical ward, *WMW* Women’s medical ward

### Type of oral DF modification

Table 3 shows that 88% of nurses modified oral DF prior to administration. There were a number of procedures used for modification, but CT/OC and mixing the medicine with juice were the most common procedures used by nurses (approximately 84%). CT/OC and giving the medicine as a powder was also used by 80.5%, adding juice to a spoonful of liquid medicine by 80%, and adding soft food (i.e., yogurt) to a spoonful of liquid medicine by 52%. Approximately 48% of nurses had opened an ampoule and mixed its contents with a drink or performed CT/OC and mixed the medicine with food (e.g., yogurt); this was the least frequent procedure performed by nurses (approximately 41%).

**Table 3:** Types of oral dosage forms modified by nurses before administration (*n *= 200)

Type of modification	Total frequency (%)
*Have you ever modified an oral DF prior administering it to a patient?*	
Yes	176 (88)
No	24 (12)
*Have you performed CT/OC and given the medicine as a powder?*	
Yes	161 (80.5)
No	39 (19.5)
*Have you performed CT/OC and mixed the contents into a drink?*	
Yes	84 (42)
No	116 (58)
*Have you performed CT/OC and mixed the contents of food (e.g., yogurt)?*	
Yes	41 (20.5)
No	159 (79.5)
*Have you ever added juice to a spoonful of liquid medicine?*	
Yes	80 (40)
No	120 (60)
*Have you ever added soft food (i.e., yogurt) to a spoonful of liquid medicine?*	
Yes	52 (26)
No	148 (74)
*Have you ever opened an ampoule and mixed its contents in a drink?*	
Yes	48 (24)
No	152 (76)

*DF* Dosage form, *CT/OC* Crushing tablets or opening capsules

### Food was mixed with medicine

Table 4 shows that 71.5% of nurses never mixed milk with medicine, and only 9% of nurses sometimes mixed milk with medicine. Regarding orange juice, 35% of nurses mixed it with medicine at some time; it seemed to be the preferable food for use in mixing with medicine, followed by strawberry juice (17.5% used it sometimes), banana (11%) and yogurt (10.5%). The least preferred liquid was chocolate (77% answered that they never used it), while water was the most commonly used liquid in mixing with medicine (41.5% answered very often). Some nurses mentioned other foods they use, such as grapefruit, juice and soup. Nurses in the NICU mentioned that they usually mix omeprazole capsules with NaHCO_3_ for neonates.

**Table 4:** Foods used to mix with medicine (*n *= 200)

Food type	Never	Rarely	Sometimes	Often	Very often
Milk	143 (71.5)	31 (15.5)	18 (9)	5 (2.5)	3 (1.5)
Orange juice	78 (39)	26 (13)	70 (35)	17 (8.5)	9 (4.5)
Yoghurt	140 (70)	30 (15)	21 (10.5)	5 (2.5)	4 (2)
Banana	144 (72)	26 (13)	22 (11)	5 (2.5)	3 (1.5)
Strawberry	126 (63)	31 (15.5)	35 (17.5)	6 (3)	2 (1)
Chocolate	154 (77)	28 (14)	13 (1.5)	3 (1.5)	2 (1)
Water	24 (12)	19 (9.5)	4 (2.5)	33 (16.5)	83 (41.5)

### Reasons for crushing medications

Table 5 illustrates the various factors contributing to the practice of CT/OC and mixing their contents with food. Nurses identify administering medications to patients with NGT as the most significant reason, with 41.5% of nurses frequently engaging in this procedure. Additionally, 41.5% of nurses occasionally resort to CT/OC when patients are unable to swallow larger tablets or capsules whole, opting to crush or open them instead. Thirty-nine percent of respondents indicated that CT/OC is sometimes employed if the patient refuses to take the medication in its original form. CT/OC is also carried out by 26.5% of nurses to mask the unpleasant sour or bitter taste of certain medications, and 24.5% employ this method to conceal the medicine from the patient.

**Table 5:** Reasons for crushing medications (*n *= 200)

The reason	Never	Rarely	Sometimes	Often	Very often
The tablet or capsule is too large to be given to the patient as a whole	43 (21.5)	25 (12.5)	83 (41.5)	25 (12.5)	24 (12)
To disguise from the patient the sour or bitter taste of the medicine	42 (21)	46 (23)	53 (26.5)	33 (16.5)	27 (13.5)
The patient will not accept the medicine as a whole tablet/capsule	33 (16.5)	24 (12)	78 (39)	38 (19)	27 (13.5)
To disguise or conceal the medicine from the patient	72 (36)	26 (13)	49 (24.5)	35 (17.5)	18 (9)
To give medicine to the patient on NGT	12 (6)	18 (9)	38 (19)	49 (24)	83 (41.5)

*NGT* Nasogastric tube

### Source of information

Table 6 shows the source of information that nurses usually use when they need to ask about drug issues regarding modification to a DF and mixing with food. 58% of nurses usually asked pharmacists, 44% asked other nurses, 38.5% chose a medical book, doctor, leaflet for drugs, and the internet equally, while 36.5% used publications of the MOH Journals and media were the least used sources of information.

**Table 6:** Source of information (*n *= 200)

Information source	Yes	No
Medical book	77 (38.5)	123 (61.5)
Other nurses	88 (44)	112 (56)
Publication of MOH	73 (36.5)	127 (63.5)
Doctors	77 (38.5)	123 (61.5)
Pharmacists	116 (58)	84 (42)
leaflet for drug	77 (38.5)	123 (61.5)
Publication of association	28 (14)	172 (86)
Journals	18 (9)	182 (91)
Internet	77 (38.5)	123 (61.5)
Media (newspaper, T.V)	18 (9)	182 (91)

*MOH* Ministry of Health

### Transparency and patient consent

Table 7 concerns the issues of transparency and patient consent before modification of the DF or mixing medication with food. Thirty-nine percent of nurses said that the patient is often aware that their medication is mixed into food, and 26.5% said that the procedure of mixing medication into food is rarely ‘carefully planned’ in the nursing notes, while 31.5% answered that sometimes the requirement for mixing the medicine into food is specified in the prescription. Regarding consent from patients and guardians before mixing drugs into food, 33% said sometimes, while 30.5% said they never asked the patient themselves. Regarding whether nurses asked the patient about the food they preferred to mix with the drug, 25% of them answered never, while 31.5% asked the patient/guardian about food the patient preferred to mix with the drug.

**Table 7:** Transparency and patient consent (*n *= 200)

The question	Never	Rarely	Sometimes	Often	Very often
Is the patient aware that their medicine is mixed into the food?	33 (16.5)	29 (14.5)	78 (39)	78 (39)	31 (15.5)
Is the procedure of mixing medicine into the food ‘carefully planned’ in the nursing notes?	36 (18)	53 (26.5)	50 (25)	26 (13)	35 (17.5)
Is the requirement of mixing the medicine into the food explicitly mentioned in the prescription?	42 (21)	41 (20.5)	63 (31.5)	28 (14)	26 (13)
Did you obtain consent from the patient’s guardian before mixing medicine into food?	43 (21.5)	36 (18)	66 (33)	27 (13.5)	28 (14)
Did you ask the patient’s guardian about the preferred food to mix with medicine?	46 (23)	41 (20.5)	63 (31.5)	26 (13)	24 (12)
Did you obtain consent from the patient before mixing the drug into food?	61 (30.5)	45 (22.5)	56 (28)	20 (10)	18 (9)
Did you ask the patients about the food they preferred to mix with medicine?	50 (25)	43 (21.5)	57 (28.5)	30 (15)	20 (10)

### Medications that are crushed and/or mixed in food

Aspirin was the most frequently crushed by nurses (44%), followed by paracetamol (33%) and atorvastatin (33%); 29% of nurses said that they usually opened omeprazole EC capsules. Azithromycin capsules were opened and mixed with food by 24% of the nurses, 29% of nurses crushed ranitidine tablets and 17.5% crushed famotidine tablets. One-quarter of them crushed frusemide and clopidogrel tablets. 22% mixed paracetamol suspension with food and 12.5% mixed azithromycin and amoxicillin/clavulanic acid suspension. Fifteen percent of nurses broke the dexamethasone ampoules to give it orally. Nurses also mentioned other medications that they often crush and mix with foods, such as alfacalcidol, calcium, propranolol, spironolactone, NSAIDs, hypnotics, carbamazepine, phenytoin, lamotrigine and phenobarbital (Table 8).

**Table 8:** List of medications that are crushed and/or mixed in food (*n *= 200)

Medication name	Yes	No
Omeprazole capsule	58 (29)	142 (71)
Atorvastatin tablet	66 (33)	134 (67)
Azithromycin capsule	48 (24)	152 (76)
Aspirin tablet	88 (44)	112 (56)
Ciprofloxacin tablet	35 (7.5)	165 (82.5)
Ampicillin	9 (4.5)	191 (95.5)
Amoxicillin tablet	16 (8)	184 (92)
Doxycycline tablet	18 (9)	182 (91)
Furosemide tablet	50 (25)	150 (75)
Bisoprolol tablet	47 (23.5)	153 (76.5)
Enalapril tablet	63 (31.5)	137 (68.5)
Clopidogrel tablet	50 (25)	150 (75)
Ranitidine tablet	58 (29	142 (71)
Famotidine tablet	35 (17.5)	165 (82.5)
Azithromycin suspension	25 (12.5)	175 (87.5)
Ibuprofen suspension	24 (12)	176 (88)
Paracetamol suspension	44 (22)	156 (78)
Dexamethasone ampoule	30 (15)	170 (85)
Diclofenac ampoule	17 (8.5)	183 (91.5)
Amoxicillin/clavulanic acid suspension	25 (12.5)	175 (87.5)
Ibuprofen tablet	36 (18)	164 (82)
Paracetamol tablet	66 (33)	134 (67)
Amlodipine tablet	52 (26)	148 (74)

### Nurses’ knowledge regarding carrying out drug dosage procedures

This study found that 35.5% of nurses did not feel sufficiently trained to carry out these practices (CT/OC and mixing it with food), and 19.5% did not know if they were qualified to carry out this practice. Furthermore, 45.5% of the respondents said they did not feel sufficiently knowledgeable in the area of drug stability. Approximately 50% of the nurses said that modifying the DF is not part of the nurse’s role or responsibilities. Regarding training courses, 65.5% of nurses mentioned that they had never taken any training courses on mixing drugs into food, 64.5% had not taken any training courses on drug stability, and 47.5% had not taken training courses on drug-food interactions. Thirty-four percent answered the question ‘Did you chick the DF before crushing the drug?’ with no, and 32% answered no according to the question ‘Did you ask the clinical pharmacist before modifying the DF?’. Moreover, 57% were not sure whether the tablets were suitable for splitting or crushing before performing those procedures. Seventy-six percent thought that if the tablets were not suitable for splitting or crushing, this information should be in the package leaflet. Half of the nurses (50%) were worried about inhaling or taking some amount of drug that they crushed. A total of 35.5% of nurses mentioned that they crushed an ER tablet, and 40.4% crushed an EC tablet (Table 9).

**Table 9:** Perceptions of respondents of their competencies and knowledge in the application of drug dosage procedures (*n *= 200)

Question^#^	Yes	No	I do not know
Do you feel sufficiently trained to carry out these procedures?	90 (45)	71 (35.5)	39 (19.5)
Do you feel sufficiently knowledgeable in the area of drug stability?	74 (37)	91 (45.5)	35 (17.5)
Do you feel sufficiently supported by your colleagues when administering medication as a comixture?	81 (40.5)	86 (43)	33 (16.5)
Do you feel sufficiently supported by your management when administering medication as a comixture?	67 (33.5)	92 (46)	41 (20.5)
Did you report every time you mix medicine with food in the nursing note?	103 (51.5)	83 (41.5)	14 (7)
Do you think modifying the DF is part of a nurse’s role or responsibility?	79 (39.5)	99 (49.5)	22 (11)
Did you take training courses on drug stability?	52 (26)	129 (64.5)	19 (9.5)
Did you take training courses on mixing drugs into food?	52 (26)	131 (65.5)	17 (8.5)
Did you take training courses on drug-food interaction?	92 (46)	95 (47.5)	13 (6.5)
Did you check the DF before crushing the medication?	123 (61.5)	67 (33.5)	10 (5)
Did you ask the clinical pharmacist before modifying the DF?	130 (65)	63 (31.5)	7 (3.5)
Sometimes I am not sure whether the tablets are suitable for splitting or crushing	113 (56.5)	70 (35)	17 (8.5)
If the tablets are not suitable for splitting or crushing, I expect to find this information in the package leaflet	145 (75.5)	34 (17)	21 (10.5)
Are you certain that the patient takes the whole amount of drug when mixed with food?	101 (50.5)	75 (37.5)	24 (12)
Did you feel worried about inhaling or taking some amount of drug while crushing it?	101 (50.5)	77 (38.5)	22 (11)
Have you crushed an ER tablet?	71 (35.5)	98 (49)	31 (15.5)
Have you crushed an EC tablet?	81 (40.5)	99 (49.5)	20 (10)
Did you check the DF before crushing the tablet?	135 (67.5)	54 (27)	11 (5.5)

*DF* Dosage form, *ER* Extended release, *EC* Enteric coated

^#^These questions were adapted from the Akram and Mullen study [12]

## Discussion

The majority of nurses (88%) frequently modified pharmaceutical DF before administration to patients, consistent with the results of a similar study carried out in Scotland [12] at 87% and in France at 81% [1]. Most nurses (80%) performed CT/OC and gave it to the patient as a powder; other common ways were mixing the medication with juice (42%) and adding juice to a spoonful of liquid medicine (40%). In a study conducted in Scotland [12], 87% of nurses gave medication as a powder after CT/OC, 96% mixed it with food and 76% mixed it with juice. This high percentage indicates the commonness of this practice that requires programmed education for nurses regarding crushing and mixing medications with foods.

Water and orange juice are commonly used for mixing. In the Scottish study from 2012 [12], the most common food was fruit yogurt or fruit juice in another study conducted in 2015 [13]. Fruit juice was used by 34% and yogurt by 34%; these were the most commonly used foods to mix with medication. In a study carried out in France [1]. Nurses used water (21% always) to mix with crushed medication or food (5% always), while in an Australian study [28], the most common food used was thickened pear juice (55.0%), followed by strawberry jam (24.0%), yogurt (6.9%) and chocolate milk (3.4%). Therefore, nurses should be aware of the characteristics of the fluid, i.e., the pH. Most fruit juices, such as orange and pineapple juices, have a pH value between 3.50 and 3.97 [29]. The chemical stability of many acid-sensitive medications is affected in that pH range. Therefore, the time between the addition of food to the medicine and the administration of the medication to the patient is considered an important factor for the clinical performance of crushed medications. Using yogurt as a vehicle to mix with crushed tablets and administer to the patient is controversial because drug dissolution may be affected. In addition, drug bioavailability depends on gastric emptying time, which is affected by food in the stomach. Studies have shown that liquids such as juice, water and milk products can lead to some major differences in gastric emptying time due to variations in solution properties, such as viscosity, osmolality, and calorie content. Although some information is available on the crushing of the tablet and the consequent effect on stability/degradation, there is considerable concern about the administration of medication via NGT [30–32]. Therefore, clinical pharmacists should be familiar with the scientific properties of foods (e.g., pH, viscosity, calorie content, and chemical composition) and common foods used by nurses to mix with medications prior to administration to better inform nursing and medical colleagues on how to assist with administration without disrupting the properties of the medication.

The most common reason for CT/OC was to give medications to patients with NGT. In a study conducted in Scotland [13], patients not accepting the medicine was the most prevalent reason. The second was the size of the tablets (36%), the bitter taste of the medication (32%), and the disguise of the medicine from patients (28%). In France [1], 67% of nurses said that they always crushed all medications and gave them together through NGT. Importantly, pharmacists and other nurses were the preferred sources of information. These findings raise the importance of pharmacists, and nurses should be aware of this drug administration struggle and be able to find a solution to help administration without affecting the chemical properties and clinical effects of the drug.

Unfortunately, more than 50% of the time, the medication being modified and mixed with food was not recorded in nursing notes or even mentioned by the prescriber, although this practice can be a reason for error in medication administration and could cause harm to the patient or failure of treatment. Administration of any medicine after alteration of the original DF by any healthcare provider is considered an off-label use of medication, and administration of medication, particularly after mixing with food or a thickening agent, could be observed as an unlawful practice [33].

Most of the time, nurses were not sure that the tablets were suitable for crushing or splitting. If the ER tablet is crushed, the amount of the medication can be released instantly, which can cause medication toxicity. Crushing EC medications can damage the coating of the drug and expose the medication directly to the acidic atmosphere in the stomach, which can irritate the lining of the stomach. Moreover, this can lead to inactivation of the drug if it is unstable in an acidic medium. Crushing these formulations (ER or EC) is a medication error that should be avoided [34, 35]. For example, omeprazole is commercially available as EC granules because it is an acid-labile medication that is inactivated by gastric acid. It is often crushed and mixed with water or sodium bicarbonate, which may decrease bioavailability and effectiveness, resulting in treatment failure [36, 37]. Furthermore, crushing of the aspirin EC tablet may cause local irritation of the stomach mucosa after oral administration [38].

In this study, we found that 7.5% of nurses usually gave ciprofloxacin to food and 9% gave doxycycline to food. Milk and yogurt affect the bioavailability of tetracycline and quinolone and lower plasma concentrations if ingested concomitantly by producing a non-absorbable insoluble chelate complex. To avoid therapeutic failure, ingestion of dairy products with ciprofloxacin or doxycycline is not recommended. Furthermore, a reduction in the dissolution rate and a prolongation of the disintegration time have been observed with concomitant ingestion of ciprofloxacin with food, caused by an increase in gastric viscosity and reduced solubility at elevated gastric pH [39, 40]. In addition, the physiological response to food intake, such as gastric acid secretion and gastric viscosity, can reduce the bioavailability of certain drugs preferentially given to an empty stomach [40].

Some drugs can be chewed, crushed, or cut (split) to aid in administration, but due to their formulation or PK properties, there is a growing list of items that are dangerous and unsafe to use in this way. One of them is antiepileptic medications, which are not preferred or limited in their use in elderly patients with swallowing problems because this medication must not be cut, crushed, or chewed [41, 42]. In our study, some nurses mentioned antiepileptic medications that they usually crush before administration, such as carbamazepine, phenytoin, lamotrigine, and phenobarbital.

## Strengths and limitations

To our knowledge, this study is one of the first to investigate the knowledge and practice of mixing drugs with food in Palestine, as well as to conduct face-to-face interviews to obtain more complete data. Furthermore, our study was conducted in six major government-run hospitals in different locations in different cities of Palestine, covering a relatively large number of hospitals.

However, the present study faced several limitations. First, the current study does not include private hospitals, and the samples were collected from government hospitals in the northern West Bank. Second, this was a cross-sectional study, so causal relationships between the scores and their related variables are difficult to prove. Third, data were obtained via a face-to-face interview that may have introduced the effects of interviewer bias [43]. Finally, in this study, a convenience sample was chosen. Selection bias can lead to a nonrepresentative sample of all nurses employed in Palestinian government hospitals or overstated and/or misguiding results [44].

## Conclusions

The results of our study show that crushing and mixing different medications in many foods is a widespread practice among nurses in Palestine, although many DFs are inappropriate for modification. Most nurses were unaware of several aspects related to mixing drugs with food. In educating nurses about conditions where crushing drugs are inappropriate or must be avoided, pharmacists may play an important role and should recommend suitable alternatives where available. To improve this practice, collaboration between nurses and pharmacists to increase pharmaceutical knowledge among nurses requires more study.

Clearly, disparities in the degrees of knowledge can be observed between nurses of different hospital departments. Structured documents that could be applied at the hospital level to crush medication and mix medications with food are needed. In addition, more courses for undergraduate nurses on medication administration and activation of the role of the clinical pharmacist in hospitals in the continuous education of nurses about medication handling and administration, as well as further investigations into inappropriate DF modification, are needed. Therefore, the results of this study are of significant value due to the following. First, develop appropriate preventive measures to minimize medication administration errors through a training course on drug-food compatibility and what medications can or cannot be mixed with food and, if so, the appropriate foods to use. Second, in hospitals, clinical pharmacists' role should be highlighted by asking them about food-drug compatibility, the chemical and physical properties of drugs that can be changed when mixed with food, and the appropriate foods to use with each drug.

## Data Availability

The datasets used and/or analyzed during the current study are available from the corresponding authors on reasonable request.

## Abbreviations

DFs: Dosage form
CT/OC: Crushing tablets or opened capsules
ER: Extended release
SR: Sustained release
MR: Modified release
Tmax: Time to maximum concentration
Cmax: Maximum plasma concentration
NGT: Nasogastric tube
IV: Intravascular
IM: Intramuscular
PD: Pharmacodynamic
PK: Pharmacokinetic
GI: Gastrointestinal
MAO: Monoaminoxidase
ICU: Intensive care unit
NICU: Neonatal intensive care unit
MMW: Men’s medical ward
WMW: Women’s medical ward
MOH: Ministry of Health
IRB: Institutional Review Board
SD: Standard deviation
IQR: Interquartile range
SPSS: Statistical Package for the Social Sciences
